# The Place of Transaxillary Access in Transcatheter Aortic Valve Implantation (TAVI) Compared to Alternative Routes—A Systematic Review Article

**DOI:** 10.31083/j.rcm2405150

**Published:** 2023-05-19

**Authors:** Andries Herremans, Dylan Thomas Stevesyns, Hicham El Jattari, Michaël Rosseel, Liesbeth Rosseel

**Affiliations:** ^1^Department of Cardiology, Algemeen Stedelijk Ziekenhuis, 9300 Aalst, Oost-Vlaanderen, Belgium; ^2^Faculty of Medicine and Health Sciences, University Gent, 9000 Gent, Oost-Vlaanderen, Belgium

**Keywords:** transcatheter aortic valve implantation, TAVI, transaxillary, aortic valve stenosis, interventional cardiology

## Abstract

**Background:**

Transfemoral transcatheter aortic valve implantation (TAVI) has proven non-inferior or superior against surgical aortic valve replacement (SAVR) for patients at high, intermediate or low surgical risk. However, transfemoral access is not always feasible in patients with severely atherosclerotic or tortuous iliofemoral arteries. For these cases, alternative access techniques have been developed, such as transcarotid, transcaval, direct aortic or transaxillary access. In recent years, growing preference towards the transaxillary access has emerged. To provide a summary of data available on transaxillary TAVI and compare this approach to other alternative access techniques.

**Methods:**

A literature search was performed in PubMed by two independent reviewers. Studies reporting the outcome of at least 10 patients who underwent transaxillary TAVI, either in case series or in comparative studies, were included in this review. Articles not reporting outcomes according to the Valve Academic Research Consortium (VARC) 1–3 definitions were excluded.

**Results:**

In total 193 records were found of which 18 were withheld for inclusion in this review. This review reports on the combined data of the 1519 patients who underwent transaxillary TAVI. Procedural success was achieved in 1203 (92.2%) of 1305 cases. Life-threatening, major, and minor bleeding occurred respectively in 4.5% (n = 50 in 1112 cases), 12.9% (n = 143 in 1112 cases) and 8.8% (n = 86 in 978 cases). Major and minor vascular complications were reported in respectively 6.6% (n = 83 in 1256 cases) and 10.0% (n = 105 in 1048 cases) of patients. 30-day mortality was 5.2% (n = 76 out of 1457 cases). At one year follow-up, the mortality rate was 1% (n = 184 out of 1082 cases). Similar 30-day and 1-year mortality is observed in studies that compare with transaxillary, transfemoral or other alternative access techniques (*p *> 0.05).

**Conclusions:**

A wide application of transaxillary access as an alternative approach for TAVI has emerged. This technique has an excellent procedural success rate up to 92.0%, with low procedural complication rates. Clinical outcome of transaxillary TAVI is comparable to the other alternative TAVI approaches. However, these conclusions are solely based on observational data.

## 1. Introduction

**Fig. 1.**
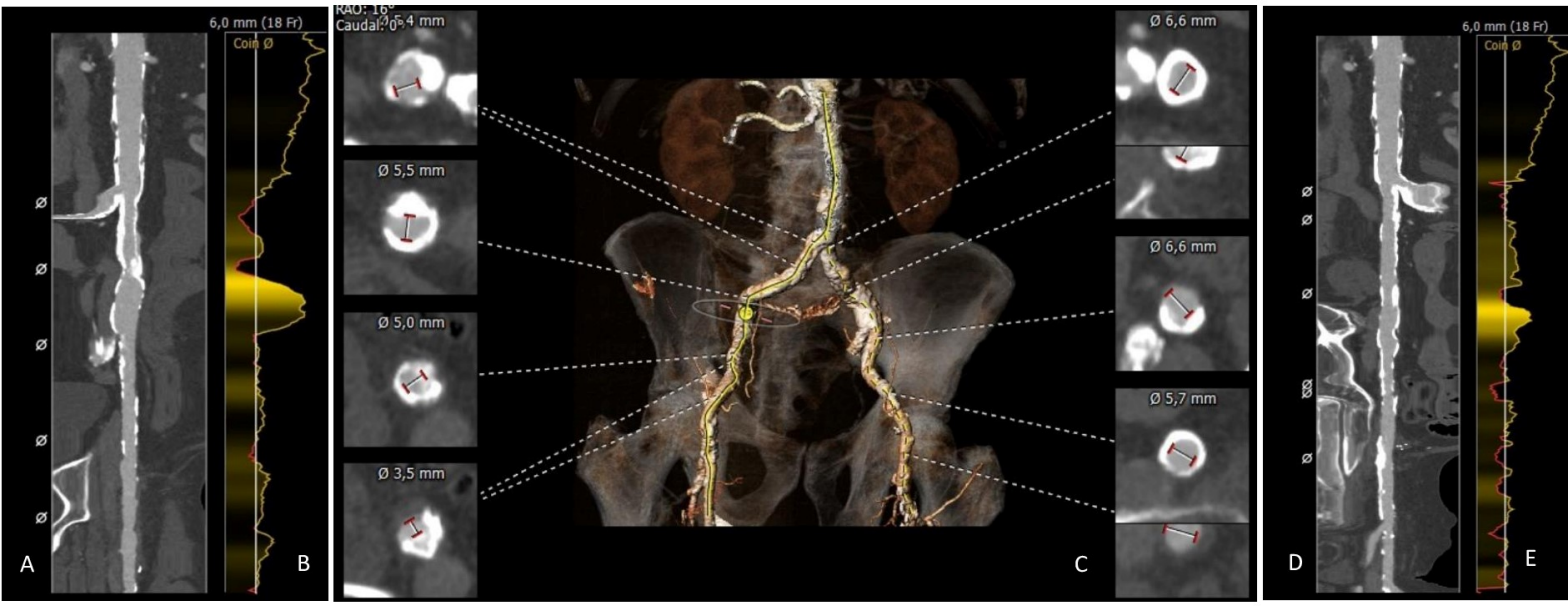
**Computed tomography images of iliofemoral access**. Stretched vessel views of the right and left iliofemoral artery (A,D) demonstrating advanced obstructive atherosclerotic disease with size of 6mm/18F minimal diameter bar set on the vessel diameter profile (B,E). En face view (C) of the iliofemoral arteries and aortic bifurcation and cross sections of most severe narrowing, with minimal lumen diameter of 3.6 and 2.9 in the right and left femoral artery, respectively.

**Fig. 2.**
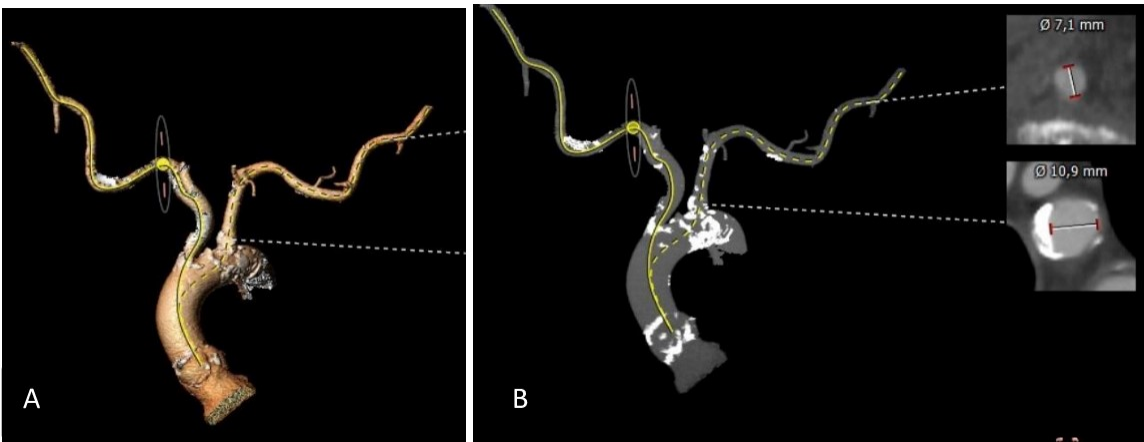
**Computed tomography images in preparation for a transaxillary TAVI procedure**. (A) Computational rendered image of aortic arch, brachiocephalic trunk and left subclavian artery. (B) Calcifications of the aortic root, arch and ascending aorta including brachiocephalic trunk and left subclavian artery. The calcification is mostly pronounced in the aortic arch and at the ostium of the left subclavian artery with cross-sectional image showing a minimal lumen diameter of 10.9 mm at this level, which is not impeding a trans-axillary access. TAVI, transcatheter aortic valve implantation.

Transcatheter aortic valve implantation (TAVI) for the treatment of symptomatic and severe aortic valve stenosis (AS) has rapidly evolved during the last decade. TAVI has proven superior or non-inferior against surgical aortic valve replacement (SAVR) for patients at high, intermediate or low surgical risk [1, 2]. Because of superior results on procedural and clinical outcome, the transfemoral technique has been the preferred access for TAVI as compared to transapical access [3, 4, 5]. Safe application of transfemoral access for TAVI is, however, precluded in patients with underlying obstructive peripheral atherosclerotic disease and/or tortuosity of the iliofemoral route (Figs. 1,2). Alternative, non-femoral and non-transapical access approaches for TAVI have thus been developed, such as a transapical, transcaval, direct aortic, transcarotid or transaxillary approach. In recent years, the transaxillary approach has gained popularity in favour of other alternative access sites [6]. This review aims to provide a summary of data available on TAVI performed through transaxillary access.

## 2. Methods

### 2.1 Search Strategy 

A literature search of the Pubmed database was performed in which the following search queries were used: (“Transcatheter Aortic Valve Replacement” OR “Transcatheter Aortic Valve Implantation”) AND (“Transaxillar*” OR “Trans-axillar*” OR “Axillar*” OR “Transubclav*” OR “Trans-subclav*”). Searches were performed by two independent investigators and all assessments of the search results were individually made. In the search process, no filters were used and all manuscripts published until December 2021 were systematically assessed.

### 2.2 Study Selection

**Fig. 3.**
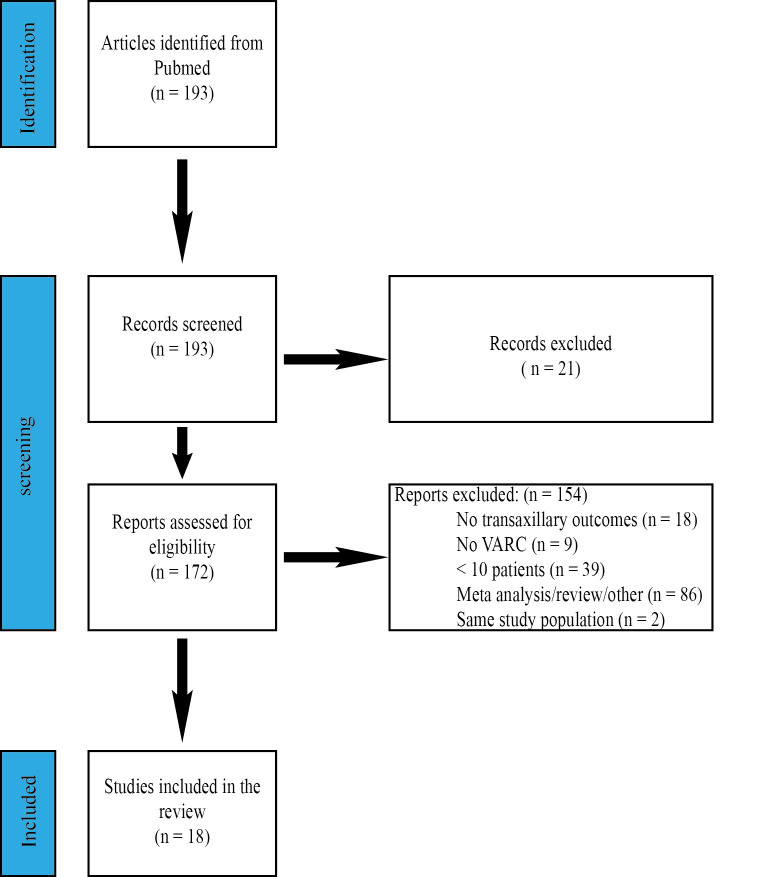
**PRISMA-flowchart detailing data extraction of the literature search**. VARC, valve academic research consortium.

The records obtained were first screened for eligibility based on title and abstract. A further exclusion was made after assessing the full text of the remaining records: meta-analyses, reviews and purely procedural descriptions were excluded, as well as case series reporting data of less than 10 patients. Studies were included when the outcomes of subsequent transaxillary TAVI procedures were reported according to the Valve Academic Research Consortium (VARC) 1–3 definitions, albeit either in case series or in comparative studies. A screening was carried out to exclude articles reporting on outcomes from same samples. Finally, the selections of both investigators were compared, and compromises were made where necessary. The study selection process is displayed according to the PRISMA-methodology in Fig. 3.

## 3. Results

### 3.1 Search Results 

In total 193 articles were withheld, of which 18 were accepted for inclusion in this review [7, 8, 9, 10, 11, 12, 13, 14, 15, 16, 17, 18, 19, 20, 21, 22, 23, 24]. Nine publications concern case series reporting on outcomes of transaxillary TAVI in either single (n = 6) [7, 8, 9, 10, 11, 12] or multi-centre (n = 3) [13, 14, 15] studies. The remainder are observational studies comparing transaxillary with one or more alternative access strategies: 5 versus transfemoral [16, 17, 18, 19, 20], 6 versus transthoracic [16, 17, 20, 21, 22, 23] and 2 versus transcarotid access [22, 24]. An overview of the included studies can be found in Table 1 (Ref. [7, 8, 9, 10, 11, 12, 13, 14, 15, 16, 17, 18, 19, 20, 21, 22, 23, 24]).

**Table 1.** **Overview of studies**.

Study	Number of transaxillary cases	Country	Study type	Self-expandable	Balloon-expandable	First- generation	Second-generation
Saia [9]	12	Italy	Prospective	12		12	
Laflamme [11]	18	Canada	Retrospective	18		18	
Deuschl [7]	12	Germany	Retrospective	12			12
Schäfer [14]	100	Germany	Retrospective				
Hysi [10]	43	France	Retrospective	16	27		43
van der Wulp [12]	362	The Netherlands	Prospective	361		311	50
van Wely [15]	45	/	Prospective	45			45
Ooms [8]	35	The Netherlands	/	35			35
Amat-Santos [13]	75	Europe and America	Retrospective	75			75
Adamo [16]	32	Italy	Prospective	32		32	
Doshi [17]	16	United Kingdom	Prospective				10
Gleason [18]	202	United States	/	202		202	
Jiménez-Quevedo [19]	191	Spain	Prospective	162	28	186	4
Zhan [20]	24	United States	Retrospective		24		24
Ciuca [21]	60	Italy	Prospective	35	24	59	
Fiorina [23]	147	Italy	Prospective	147		147	
Damluji [22]	17	France/United States	Retrospective	17		17	
Debry [24]	128	France	Prospective			37	91

First-generation self-expanding devices are: Medtronic Evolut Corevalve and Edwards Sapien-XT. Next generation: Medtronic Evolut en Edwards Sapien 3.

Two of the studies comparing transaxillary and transfemoral access include propensity matched cohorts [18, 19]. No randomised controlled trials are available in this field.

### 3.2 Transaxillary TAVI: Outcomes 

#### 3.2.1 Clinical Characteristics

In total, data of 1519 transaxillary cases was reported in the 18 included studies [7, 8, 9, 10, 11, 12, 13, 14, 15, 16, 17, 18, 19, 20, 21, 22, 23, 24]. The mean age of all transaxillary access cases was 81.1 ± 1.8 years, and 58.7% ± 12.4% were male. The Society of Thoracic Surgeons (STS) scores were reported in 13 studies with a mean STS score of 7.0% ± 2.2% [7, 8, 10, 13, 14, 15, 16, 18, 20, 21, 22, 23, 24]. The LogisticEuro-II scores were reported in 12 studies and had a mean of 18.8% ± 5.2% [7, 10, 11, 12, 14, 15, 16, 17, 18, 19, 21, 23]. Left ventricular ejection fraction (LVEF) was reported in 13 studies and has a total mean of 53.4% ± 4.5% [7, 8, 9, 10, 13, 14, 16, 19, 20, 21, 22, 23, 24]. Most reported comorbidities were arterial hypertension, coronary artery disease and peripheral vascular disease. Further patient characteristics are shown in Table 2.

**Table 2.** **Patient characteristics**.

Parameter	Unit	Mean (standard deviation) or % (n/N)
Age	years	81.1 ± 1.8
Gender (male)	%	58.7 ± 12.4
BMI	kg/$m^{2}$	26.3 ± 1.42
STS score	%	7.0 ± 2.2
Euroscore	%	18.8 ± 5.2
NYHA III/IV	% (n/N)	79.3 (971/1225)
AHT	% (n/N)	83.0 (611/736)
DM	% (n/N)	37.5 (510/1360)
AFib	% (n/N)	34.1 (405/1188)
CKD	% (n/N)	34.9 (226/648)
PVD	% (n/N)	55.2 (663/1201)
CAD	% (n/N)	60.1 (758/1261)
prior CABG	% (n/N)	16.4 (242/1474)
prior SAVR	% (n/N)	5.61 (29/517)
prior stroke/TIA	% (n/N)	19.7 (261/1328)
prior PPM	% (n/N)	10.6 (122/1150)
LVEF	%	53.4 ± 4.5

BMI, Body Mass Index; STS, Society of Thoracic Surgeons; NYHA, New York Heart Association; AHT, Arterial hypertension; DM, Diabetes mellitus; AFib, Atrial fibrillation; CKD, Chronic Kidney Disease; PVD, Peripheral vascular disease; CAD, Coronary artery disease; CABG, Coronary Artery Bypass Grafting; SAVR, Surgical Aortic Valve Replacement; TIA, transient ischemic attack; PPM, Permanent Pacemaker; LVEF, Left Ventricle Ejection Fraction.

#### 3.2.2 Procedural Characteristics

Transaxillary TAVI was performed through direct percutaneous access in 258 cases (16.9%) while in 926 cases (60.5%) surgical cutdown was performed. The left axillary artery was predominantly used (89.0%, n = 936 in 1034 cases). The most frequently used transcatheter heart valves (THV) were self-expanding devices (91.9%, 1169 in 1272 cases) versus (8.1%, n = 103 in 1272 cases) balloon-expandable devices. The great majority were treated with first-generation devices (72.4%, 1021 in 1410 cases). Further details of procedural characteristics are shown in Tables 1,3.

**Table 3.** **Procedural characteristics in transaxillary TAVI**.

Parameter	% (n/N)
Local anaesthesia	21.7 (286/1316)
Direct Percutaneous	21.6 (258/1184)
Left sided access	89.0 (1034/1162)
Pre-dilatation	60.0 (373/622)
Post dilatation	23.3 (193/828)
Self-expandable devices	91.9 (1169/1272)
First-generation devices	72.4 (1021/1410)

TAVI, transcatheter aortic valve implantation.

#### 3.2.3 Procedural Outcomes

Overall procedural success was achieved in 1203 (92.2%) of 1305 cases. Life-threatening, major, and minor bleeding occurred respectively in 4.5% (n = 50 in 1112 cases), 12.9% (n = 143 in 1112 cases) and 8.8% (n = 86 in 978 cases). Major and minor vascular complications were reported in respectively 6.6% (n = 83 in 1256 cases) and 10.0% (n = 105 in 1048 cases) of patients. Stroke occurred in 3.2% (n = 48 in 1508 cases), myocardial infarction (MI) in 2.2% (n = 25 in 1148 cases) and acute kidney injury (AKI) in 10.4% (n = 124 of 1190 cases) of cases. The most frequently observed VARC-defined endpoints were the implantation of a new permanent pacemaker (PPM), which was seen in 18.3% (n = 261 in 1435 cases). Other procedural outcomes are summarised in Table 4.

**Table 4.** **Procedural outcomes**.

Parameter	% (n/N)
procedural success	92.2 (1203/1305)
Procedural mortality	1.9 (17/898)
AR ≥ moderate	5.0 (76/1519)
Life threatening bleeding	4.5 (50/1112)
Major bleeding	12.9 (143/1112)
Minor bleeding	8.8 (86/978)
Major vascular complications	6.6 (83/1256)
Minor vascular complications	10.0 (105/1048)
AKI	10.4 (124/1190)
Stroke	3.2 (48/1508)
PPM	18.3 (261/1435)
Surgical conversion	0.9 (8/925)
Length of hospital stay (days)	7.68 ± 2.5

AKI, Acute Kidney injury; PPM, Permanent Pacemaker; AR, aortic regurgitation.

#### 3.2.4 Mid-Term Outcome

30-day mortality was reported in 17 out of 18 studies and was 5.2% (n = 76 out of 1457 cases) [7, 8, 10, 11, 12, 13, 14, 15, 16, 17, 18, 19, 20, 21, 22, 23, 24]. At one year follow-up, there was a mortality rate of 17% (n = 184 out of 1082 cases). Cerebrovascular incidents—i.e., any stroke or transient ischemic attack (TIA)—occurred in 3.2% (n = 48 out of 1469 cases) of the patients undergoing transaxillary TAVI. Only in a few manuscripts the severity of the stroke was indicated: incidence of major stroke was 1.9% (n = 12 out of 636 cases), while that of minor stroke was 1.6% (n = 10 out of 636 cases). Further details of 30-day and one year follow up can be found in Table 5.

**Table 5.** **Clinical outcomes at 30 days and 1 year**.

Parameters	30-day	1-year
	% (n/N)	% (n/N)
Mortality	5.2 (76/1457)	17.0 (184/1082)
Stroke/TIA	3.2 (48/1469)	10.4 (31/297)
MI	2.1 (25/1184)	2.3 (6/265)
Repeated intervention for valve related dysfunction	2.9 (33/1149)	1.5 (3/202)
New PPM	17.5 (261/1490)	17.7 (47/265)
AVA (${\mathrm{cm}}^{2}$) ± standard deviation	1.79 ± 0.17	1.86 ± 0.12
PVL ≥2	5.0 (76/1519)	4.6 (17/372)

TIA, transient ischemic attack; MI, Myocardial infarction; PPM, Permanent Pacemaker; AVA, aortic valve area; PVL, Para Valvular Leak.

### 3.3 Transaxillary TAVI Compared to other Access Technique

Nine of the articles included in this review compared the results of transaxillary TAVI with one or more alternative access techniques, such as a transfemoral (n = 5) [16, 17, 18, 19, 20], transthoracic (n = 6) [16, 17, 20, 21, 22, 23] and transcarotid (n = 2) [22, 24].

#### 3.3.1 Transaxillary vs Transfemoral Access

Five studies compared outcomes of transfemoral access to transaxillary access, among them 2 studies compared propensity score matched groups [18, 19]. One of these matched cohort studies (n = 189) reported a significantly higher 30-day mortality rate in the transaxillary group as compared to the transfemoral group (7.9% vs 4.3%, *p* = 0.04) [19]. However, there was no difference for 30-day mortality in the other matched cohort trial (5.5% vs 5.9%, *p* = 0.83), neither was there in the remaining 3 studies (6.4% vs 5.3%, *p* = 0.50; 0.0% vs 4.0%, *p* = 0.43; and 0.0% vs 2.0%, *p* = 0.51) [16, 17, 18, 20]. Only 2 studies reported on 1-year mortality, showing no significant difference between both groups (23.3% vs 24.8%, *p* = 0.70 and 25.8% vs 16.2%, *p* = 0.33) [16, 18]. For stroke, life-threatening and major bleeding, major vascular complications and AKI, no significant differences were observed between the transaxillary and transfemoral groups as shown in **Supplementary Table 1**. A significantly higher occurrence of MI was reported in the transaxillary group as compared to transfemoral TAVI in one propensity score matched studies (3.6% vs 0.8%, *p* = 0.001) [19]. In the same propensity matched study as well as in another comparative study, a significantly higher need for PPM implantation has been described in the transaxillary group (21.0% vs 15.0%, *p* = 0.03 after propensity score matching and 38.0% vs 6.0%, *p *< 0.01 respectively) [17, 19]. In the remainder there was no significant difference in PPM rate. An overview of clinical outcomes in the different studies comparing transaxillary versus alternative access are shown in the **Supplementary Table 1**.

#### 3.3.2 Transaxillary vs Transthoracic Access

Procedural outcomes for TAVI performed through transapical or direct aortic access have been reported as being similar [6]. Therefore, in this review, the articles reporting on the comparison of transaxillary with either transapical or direct aortic TAVI have been taken together as a transthoracic group.

Regarding the 30-day mortality, all 6 studies show a trend being lower in the transaxillary compared to the transthoracic group, albeit without any reported significance (all *p *> 0.05) [16, 17, 20, 21, 22, 23]. Also, no significant difference was reported in either of the 2 articles that report on 1-year mortality (25.0% vs 18.2%, *p* = 0.33; 11.8% vs 14.3%, *p *> 0.05) [16, 22]. However, statistical significant differences in the comparison of life-threatening as well as major bleeding rates were found in one study, showing a lower occurrence of both outcomes in the transaxillary groups (8.3% vs 15.5%, *p *< 0.001 and 3.3% vs 23.9%, *p *< 0.001) [21]. Contrarily, PPM implantation occurred more frequently after transaxillary TAVI in most studies reporting on this outcome, even reaching significance in 3 analyses (38.0% vs 4.0%, *p* = 0.001; 27.1% vs 5.6%, *p *< 0.001; and 34.0% vs 13.0%, *p* = 0.02) [17, 21, 23]. The incidence of AKI after transaxillary TAVI has been reported to be lower as compared to transthoracic TAVI in all 5 studies reporting on this outcome, with only one of these reporting a significant difference (22.0% vs 36.0%, *p* = 0.02) [23]. Regarding the occurrence of post-procedural stroke, major vascular complications and MI, no significant difference between the two groups has been reported and no clear trends became apparent when comparing the studies, as shown in **Supplementary Table 1**.

#### 3.3.3 Transaxillary vs Transcarotid Access

Two articles reported on the results of TAVI through transaxillary compared to transcarotid access. This resulted for one study in a comparison of 113 transaxillary and 201 transcarotid cases, and for the other 17 versus 43 patients respectively [22, 24]. In neither study any statistically significant differences in clinical outcomes were observed (**Supplementary Table 1**) [22, 24].

## 4. Discussion

We conducted a review of the literature concerning transaxillary access as alternative route for TAVI. We conclude the following: (1) over the recent years, a wide application of the transaxillary approach for TAVI has developed; (2) this technique has an excellent procedural success rate up to 92%, with low procedural complication rates; and (3) clinical outcomes of transaxillary TAVI appear to be comparable to other alternative access approaches for TAVI.

Data from the STS and American College of Cardiology (ACC) Transcatheter Valve Therapy (TVT) Registry, a large database of TAVI procedures performed in the United States nicely demonstrate the evolution of TAVI access over the years. Transfemoral access was only being used in 57.1% of TAVI cases in 2013 [6, 25]. In the early TAVI years, when the insertion profiles of transcatheter heart valves (THV) were still much larger, a significant amount of procedures were performed via the—Food and Drug Administration (FDA) approved—transapical access reaching up to 34.2% in 2013, and the alternative direct aortic access technique increased up to 8.7% in 2014. However, in the following years, unfavourable data on transapical access, the availability of next-generation devices with lower insertion profiles, and development of other, less invasive, alternative access techniques, has led to an enormous downfall of transapical and direct aortic TAVI procedures (0.5% and 0.3% in 2019, respectively), while overall transfemoral approach increased up to 95.3% of TAVI cases in 2019 [25]. During these years, the transaxillary approach has shown a remarkable rise in popularity. On the other hand, the transcaval access became a newly adopted technique, albeit with limited use (121 cases in 2019), while the transcarotid approach became less common. Transaxillary access became the most commonly employed alternative access technique, accounting for 1816 cases, or 2.5% of all TAVI cases performed in 2019 in the United States [6, 25].

The population of patients who are selected to undergo a TAVI through transaxillary access are generally relatively old with multiple comorbidities and STS-scores ranging between intermediate to high surgical risk. Despite these comorbidities, which are often associated with higher morbidity and mortality after TAVI, procedural success and post-procedural outcomes of transaxillary TAVI appear to be favourable with procedural success of 92% and low post-procedural complications such as MI and life-threatening bleeding [3]. Also, 30-day and 1-year mortality rates of 5% and 17%, respectively, are in line with results of the TVT registry data [25]. On the other hand, major bleeding and vascular complications are more common in transaxillary access cases, often due to closure device failure and/or difficulty compressing the axillary artery. Importantly, these latter do not seem to be associated with worse clinical outcome and are often manageable with percutaneous techniques such as the use of covered stents.

The number of patients suffering from stroke after transaxillary TAVI appears to be low based on our collected data, however, according to larger registries such as the STS-ACC TVT Registry report stroke rates up to 6% after transaxillary TAVI [6]. These results are probably caused by the relatively large amount of studies with small study population in our manuscript. After all, 1 in 3 included studies report on a study population smaller than 30 patients, none of which suffer from post-procedural stroke [7, 9, 10, 11, 12, 14]. Therefore, a caveat must be made concerning post-procedural stroke rates after transaxillary TAVI, as future, larger registries may show higher occurrence of cerebrovascular accident (CVA).

The most frequently occurring VARC-defined outcome in this analysis was the new PPM implantation rate, which reached up to 18% when all data was combined. This higher PPM rate is most probably attributable to the use of mainly first-generation THV devices, that were none repositionable, and were implanted without the adoption of contemporary implantation techniques (e.g., cusp overlap view) that are developed to aim for high implant to reduce the risk for new PPM.

No significant differences in procedural success, 30-day and 1-year mortality were demonstrated in the different studies comparing transaxillary and transfemoral access in this review [16, 17, 18, 19, 20]. The same can be found for other procedural outcomes such as stroke, life-threatening and major bleeding, major vascular complications and AKI with only one of the included studies reporting a significantly higher incidence of MI. Six meta-analyses that compare transaxillary versus transfemoral access are available [3, 4, 26, 27, 28, 29]. In neither of these meta-analyses, a significant difference in 30-day mortality was shown. No significant difference was observed for mortality at 1 year and 1,6 years respectively in two available meta-analyses that report on mid-term results [4, 28]. A third, however, reported a higher midterm mortality (period unspecified) in transaxillary compared to transfemoral cases [3]. This may be explained by the significantly higher midterm stroke rate in the transaxillary group reported in this meta-analysis. Contrarily, in none of the other 5 meta-analyses a difference could be found for stroke rates between transaxillary and transfemoral TAVI. Furthermore there were no significant differences found in all bleeding events in 5 of the meta-analyses [3, 4, 26, 28, 29] while in 2 meta-analyses a significant lower vascular complication rate was seen in the transaxillary group as compared to the transfemoral group [26, 27]. In the remaining 4 meta-analyses there was no difference in vascular complications noted [3, 4, 28, 29]. Fewer AKI was seen in the transaxillary group compared to the transfemoral approach in two of these meta-analysis [3, 29]. The reason for this is unclear, but one hypothesis is that a more direct THV positioning (less ‘slack’) in the annulus due to the shorter route with the transaxillary access might lead to less contrast use.

Whether to decide if transaxillary TAVI is superior to any of the other alternative access techniques, can only be based on data from observational registries, matched cohort retrospective studies and meta-analyses. Up to now, no studies have investigated alternative access techniques in a randomized manner. Patients—with typically a high burden of peripheral atherosclerotic disease—who are not eligible for transfemoral TAVI, often qualify for only limited number of the possible alternative approaches. Furthermore, due to a learning curve aspect, with improvement of outcomes when experience increases, operators tend to gain experience in a limited amount of alternative access approaches. Both imply that the set-up of randomized trials for this matter remains difficult.

### Evolutions

Some interesting ongoing evolutions in the optimization of the technique should be noted, most strikingly the development of a percutaneous technique. Initially, transaxillary TAVI was performed via arteriotomy through surgical cut-down. Already in 2012, Schäfer [30] demonstrated a percutaneous method for delivering transaxillary TAVI in 24 patients, which they called the “Hamburg Sankt Georg approach”, with excellent results. A subsequent report describing the outcomes of this percutaneous technique on 100 consecutive patients showed similar results, as well as the presence of a learning curve demonstrating improved outcome with increasing experience [14]. The results and outcomes of percutaneous versus surgical access for TAVI appear to be similar between the two groups concerning mortality, stroke and vascular complications, while major bleeding complications were shown to occur more frequently in the surgical access group as compared to the percutaneous group [6, 31]. A simplification of the transfemoral TAVI procedure has already proven to lead to better results with shorter hospital stay. Also here, seemingly beneficial effects of percutaneous over surgical access techniques are observed. For example, with direct percutaneous access the use of general anaesthesia can be omitted [32]. In a case series of Ooms [8] favourable outcomes for transaxillary approach were reported, demonstrating transaxillary TAVI under local anaesthesia to be a safe and feasible alternative to generalised anaesthesia. Possible advantages brought forwards by the authors include a more favourable recovery time, decreased risk for infections and delirium, and, real-time monitoring of cerebrovascular events during the procedure.

Specific techniques of local anaesthesia, such as pectoral-1 and -2 block, superficial cervical plexus block and interscalene block have been described as alternative approaches to perform transaxillary TAVI procedures [33, 34, 35].

## 5. Study Limitations

In this review, only observational studies were included because of the absence of randomised controlled trials in the area. Furthermore, patient characteristics may differ between the groups, making real head-to-head comparison difficult.

Even though we only included manuscripts using VARC-definitions, the comparison of the results and outcomes was hampered by the omitting of certain endpoints. Moreover, regular revisions of the VARC-definitions—with a recent renewal in VARC-3—complicates the comparison of results across different time periods.

## 6. Conclusions

Transaxillary access is considered a feasible and safe alternative approach for TAVI in patients not eligible for implantation of a transcatheter aortic valve by transfemoral access. With increasing experience and subsequent refinement of the technique, transaxillary access may gain further evidence and popularity so that it may further solidify its position as the first-choice alternative to transfemoral TAVI emplacement.

## Supplementary Material

Supplementary material associated with this article can be found, in the online version, at https://doi.org/10.31083/j.rcm2405150.
