# Supplementary material for: The Place of Transaxillary Access in Transcatheter Aortic Valve Implantation (TAVI) Compared to Alternative Routes—A Systematic Review Article
Source: Rev Cardiovasc Med. 2023 May 19;24(5):150. doi: 10.31083/j.rcm2405150 (PMC11273050; doi:10.31083/j.rcm2405150)
Supplement: Supplementary file 1 [file 2153-8174-24-5-150-s1.zip › Supplementary Material.docx]

| patient characteristics | Total number of patients | TA (N) vs. X (N) | 30-day mortality | 1-year mortality | Life-threatening bleeding | Major bleeding | minor bleeding | Major vascular complication | Minor Vascular complication | AKI | MI | Stroke | New PPM |
| --- | --- | --- | --- | --- | --- | --- | --- | --- | --- | --- | --- | --- | --- |
| Saia [9] | 12 |  | / | / | / | / | / | / | / | 6 | / | / | / |
| Laflamme [11] | 18 |  | 0 | 2 | 0 | 1 | 2 | 0 | 1 | 1 | 0 | 0 | 7 |
| Deuschl [7] | 12 |  | 0 | 0 | 0 | 1 | 1 | 0 | 1 | 0 | 0 | 0 | 0 |
| Schäfer [14] | 100 |  | 6 | 15 | 3 | 0 | 6 | 0 | 11 | 3 | 0 | 1 | 23 |
| Hysi [10] | 43 |  | 3 | 6 | / | / | / | / | / | / | / | 1 | 8 |
| van der Wulp [12] | 362 |  | 19 | 69 | 6 | 37 | 38 | 17 | 53 | 5 | 10 | 5 | 41 |
| van Wely [15] | 45 |  | 1 | 2 | 2 | 5 | 9 | 5 | 11 | 0 | 0 | 5 | 2 |
| Ooms [8] | 35 |  | 1 | / | 3 | 2 | 3 | 6 | 0 | 3 | 0 | 2 | 5 |
| Amat-Santos [13] | 75 |  | 2 | 6 | 2 | 5 | 7 | 11 | 6 | / | 2 | 1 | 6 |
| Adamo [16] | 32 | TA (32) vs TF (246) vs TT (44) | 6.4 % vs 5.2% vs 9.3%;  P = 0.51 | 25.8 % vs 16.3 % vs 18.6 %;  P = 0.33 | 0.0 % vs 1.2 % vs 2.3 %;  P = 0.54 | 6.2 % vs 1.6 % vs 0.0 %;  P = 0.17 | 3.1 % vs 2.0 % vs 2.3 %;  P = 0.52 | 0.0 % vs 2.8 % vs 0.0 %;  P = 0.62 | 0.0 % vs 5.3 % vs 0.0 %;  P = 0.03 | 12.5 % vs 29.7 % vs 29.5 %;  P = 0.62 | 0.0 % vs 0.8 % vs 2.3 %;  P = 0.65 | 3.1 % vs 2.4% vs 2.3 %; P = 0.89 | 15.6 % vs 13.8 % vs 11.4 %; P = 0.87 |
| Doshi [17] | 16 | TA (16) vs TF (347) vs TT (45) | 0.0% vs 4.0% vs 11.0 %;  PTFvsTA = 0.43. PTAvsTT = 0.16 | / | / | / | / | 0.0 % vs  4.0 % vs  4.0 %;  P TFvsTA = 0.43 PTAvsTT = 0.39 | / | / | 0.0 % vs 1.0 % vs 2.0 %;  P TFvsTA = 0.63 PTAvsTT = 0.55 | 0.0 % vs 0.3 % vs 4.0 %;  P TFvsTA = 0.83. PTAvsTT = 0.39 | 38.0 % vs 6.0 % vs 4.0 %;  P TFvsTA < 0.001. PTAvsTT = 0.001 |
| Gleason [18] | 202 | TA (202) vs TF (202)* | 8.4 % vs 5.9 %;  P = 0.83 | 23.3 % vs 24.8 %;  P = 0.70 | 11.4% vs 10.4 %;  P = 0.74 | 27.8 % vs 22.8 %;  P = 0.26 | / | 11.9 % vs 10.4 %;  P = 0.64 | / | 10.0 % vs 14.4 %;  P = 0.18 | 1.5 % vs 1.0 %;  P = 0.65 | 7.5 % vs 5.0 %;  P = 0.30 | 19.5 % vs 26.4 %;  P = 0.09 |
| Jiménez-Quevedo [19] | 191 | TA (191) vs TF (6412)* | 7.9 % vs 4.3 %;  P = 0.39 | / | / | / | / | / | / | / | 3.6% vs 0.8%;  P = 0.01 | 4.2% vs 2.0%;  P = 0.19 | 20.9 % vs 15.2 %;  P = 0.03 |
| Zhan [20] | 24 | TA (24) vs TF (100) vs TT (20) | 0.0 % vs 2.0% vs 5.0%;  P = 0.51 | / | 0.0 % vs 0.0 % vs 0.0 %  P = / | 0.0 % vs 3.0 % vs 0.0 %;  P = 0.51 | 4.2 % vs 2.0 % vs 0.0 %;  P = 0.63 | 4.2 % vs  3.0 % vs  5.0 %;  P = 0.89 | 0.0 % vs  3.0 % vs  0.0 %;  P = 0.51 | 13.0 % vs 5.1 % vs 23.5 %;  P = 0.03 | 0.0 % vs  0.0 % vs  0.0 %  P = / | 0.0 % vs 2.0 % vs 5.0 %;  P = 0.51 | 16.7 % vs 8.9 % vs 23.5 %;  P = 0.13 |
| Ciuca [21] | 60 | TA (60) vs TT (142) | 1.7 % vs 8.5 %;  P = 0.06 | / | 8.3 % vs 15.5 %;  P < 0.001 | 3.3 % vs 23.9 %;  P = / | / vs  14.1 %;  P = / | 3.3 % vs 6.3 %;  P = 0.44 | 6.7% vs 3.5%;  P = / | 35.1 % vs 41.1 %;  P = 0.27 | 3.3 % vs 3.5 %;  P = 0.93 | 3.3 % vs 1.4 %;  P = 0.34 | 27.1 % vs 5.6;  P < 0.001 |
| Fiorina [23] | 147 | TA (147) vs TT (95) | 5.0 % vs 9.0 %;  P = 0.5 | / | 4.0 % vs  5.0 %;  P = 0.7 | 22.0 % vs 15.0 %;  P = 0.17 | 9.5 % vs  18.0 %;  P = 0.05 | 4.0 % vs 3.2 %;  P = 0.67 | 10 % vs  2.1 %;  P = 0.02 | 22.0 % vs  36.0 %;  P = 0.02 | / | 1.4 % vs 1.1 %;  P = 0.81 | 34.0 % vs 13.0 %;  P = 0.017 |
| Damluji [22] | 17 | TA (17) vs TT (112) vs TC (43) | 0.0 % vs 10.7 % vs 8.0 %; P = 0.08 | 17.0 % vs 14.3 % vs 8.0 %;  P = 0.26 | / | / | / | / | / | 0.0 % vs 6.3 % vs  2.0 %;  P = 0.37 | / | 0.0 % vs 5.3 % vs 2.0 %;  P = 0.51 | / |
| Debry [24] | 128 | TA (113) vs TC (201)* | 5.5 % vs 4.5 %; P = 0.71 | 16.1 % vs 19.1 %;  P = 0.62 | 3.6 % vs 5.7 %;  P = 0.44 | | 2.7 % vs 9.3 %; P = 0.04 | 9.0 % vs  8.5 %;  P = 0.70 | 2.7 % vs  7.0 %;  P = 0.08 | 22.5 % vs 13.5 %;  P = 0.10 | / | 3.2 % vs 6.8 %;  P = 0.31 | 19.5 % vs 19 %;  P = 0.97 |
